# Does food biodiversity protect against malnutrition and favour the resilience to climate change-related events in Amazon Indigenous communities? A protocol for a mixed methods study

**DOI:** 10.12688/wellcomeopenres.18235.2

**Published:** 2023-06-26

**Authors:** Carol Zavaleta-Cortijo, Janet Cade, James Ford, Darren C. Greenwood, Cesar Carcamo, Rosa Silvera-Ccallo, Connie Fernandez-Neyra, Guillermo Lancha-Rucoba, Manuel Pizango-Tangoa, Rogelia Pizango-Inuma, Junior Chanchari-Huiñapi, Jorge Velez-Quevedo, Nerita Inuma-Tangoa, Teresita Antazu, Marianella Miranda-Cuadros, Juan Pablo Aparco, Pedro Aro-Guardia, Manuela Verastegui, Valeria Morales-Ancajima, Tiana Bressan, J. Jaime Miranda

**Affiliations:** 1 Unidad de Ciudadanía Intercultural y Salud Indígena, Facultad de Salud Pública y Administración,, Universidad Peruana Cayetano Heredia, San Martín de Porres, Lima, 15102, Peru; 2 Nutritional Epidemiology Group, School of Food Science and Nutrition, University of Leeds, Leeds, LS 2 9JT, UK; 3 Priestley International Centre for Climate, University of Leeds, Leeds, LS 2 9JT, UK; 4 School of Medicine, University of Leeds, Clarendon Way, Leeds, LS2 9NL, UK; 5 Facultad de Salud Pública y Administración, Universidad Peruana Cayetano Heredia, San Martín de Porres, Lima, 15102, Peru; 6 Hospital Santa Gema Yurimaguas, Yurimaguas, Loreto, Peru; 7 Pueblo Indígena Shawi, Yurimaguas, Loreto, Peru; 8 Comunidad de 10 de Agosto, Pueblo Indígena Shawi, Balsapuerto, Loreto, Peru; 9 Comunidad de Nuevo Progreso, Pueblo Indígena Shawi, Balsapuerto, Loreto, Peru; 10 Taller Verde, Caserio San Luis s/n , Carretera Munichis, San Rafael, Yurimaguas, Peru; 11 Comunidad de Palmiche, Pueblo Indígena Shawi, cuenca del río Sillay, Loreto, Peru; 12 Programa Mujer, Asociación Interétnica de Desarrollo de la Selva Peruana (AIDESEP), La Victoria, Lima, 15034, Peru; 13 Centro Nacional de Alimentación y Nutrición, Instituto Nacional de Salud, Jesús María, Lima, 15072, Peru; 14 Centro de Hemoterapia y Banco de Sangre, Hospital Nacional Cayetano Heredia, San Martín de Porres, Lima, 15102, Peru; 15 Infectious Diseases Research Laboratory of the LID, Faculty of Sciences and Philosophy, Universidad Peruana Cayetano Heredia, San Martín de Porres, Lima, 15102, Peru; 16 Department of Engineering, University of Guelph, Guelph, ON, N1G 2W1, Canada; 17 Facultad de Medicina, Universidad Peruana Cayetano Heredia, Miraflores, Lima, 15074, Peru

**Keywords:** Nutrition, anaemia, biodiversity, Amazonia, climate resilience, Indigenous people, food intake

## Abstract

**Background** : Undernutrition is projected to be a major consequence of climate change. Biodiversity could enhance climate change resilience by improving nutritional outcomes and providing healthy food resources during and/or after climate-related events. For Indigenous populations who currently base their diet on local biodiversity, rapid climate changes may affect their ability to produce, access or gather food and consequently impact their nutritional status. There is a knowledge gap regarding whether nutritional status among Indigenous populations is better among those who consume a diet with greater biodiversity than those who have a diet with low biodiversity.

**Objective** : This study aims to investigate the role of food biodiversity (FBD) in nutritional resilience to extreme flooding events of Shawi Amazon Indigenous adults living in Peruvian communities that have experienced extreme floods in the past five years.

**Methods** : This study will use a mixed-method sequential explanatory design. The quantitative component includes a cross-sectional survey to assess the association between food biodiversity (FBD) and the prevalence of anaemia in adults aged 15 to 60 years old (n=365). Anaemia will be evaluated using blood hemoglobin and serum ferritin. FBD will be measured with a food frequency questionnaire and a 24-hour dietary recall. Soil-transmitted helminth infections, malaria, and inflammatory biomarkers will also be evaluated. The qualitative component will include a community-based participatory approach to investigate the role of FBD in the responses to extreme floods. Male (n=14) and female (n=14) participants, previously identified in the quantitative phase with high and low levels of FBD, will be invited to participate in a Photovoice activity and semi-structured interviews. A analytical framework for climate change resilience will be used to integrate the data.

**Discussion** : Findings will be integrated to identify nutritional resilience indicators that can inform adaptative interventions to changing climatic conditions in the Amazon and that respect Indigenous worldviews

## Introduction

Undernutrition is projected to be a major consequence of climate change ^1– 3^. The World Health Organization (WHO) has estimated that climate change will be the cause of two million additional deaths due to undernutrition in children between 2030 and 2050 ^4^. It is anticipated that populations that are already food insecure will be among the most adversely affected by the impact of climate change on food systems ^5^. Biodiversity could enhance the resilience of food systems to climate change by improving nutritional outcomes, providing healthy, local food sources during and after climate-related risks, and allowing local populations to develop a more sustainable food system ^6– 8^. What is unclear, however, is *how* food biodiversity (FBD) is linked through food intake to human nutritional status ^9, 10^ and impacted by climatic risks and individual and household characteristics ^11^. FBD is understood as all edible animal and vegetable species, including their products such as eggs and honey, collected from the forest and produced on farms and consumed as part of the usual diet ^12– 14^.

Multiple studies have reported that a biodiverse diet offers high concentrations of macro and micronutrients, implying a higher nutritional status is favored ^14, 15^. In a diverse diet, wild food species such as fruit, fish, bushmeat, mushrooms, and arthropods may offer an appropriate food substitute when crop yields fail due to unpredictable seasons ^7, 16^. Food species deemed inedible in Western diets, like insects, are highly appreciated and consumed by approximately two billion people worldwide ^17^. Wild and semi-domesticated fruits could be a rich source of micronutrients for populations with poor nutritional status in highly biodiverse regions ^13, 18, 19^. A better understanding of the diversity of food species and their nutritional composition is necessary to clarify the role of biodiversity in the human diet and inform the response to climate change impacts on food and nutritional security ^8^. Biodiversity may enhance the bioavailability of key micronutrients for populations where the diet is mainly plant-based ^20^. For example, a combination of nutrients can offset the inhibitory effects of phytates on non-haem iron absorption ^21^. However, there is a gap of knowledge regarding whether the nutritional status is better in those who consume a diet with a greater number of edible species compared to others who have a diet with low biodiversity.

Over the past two decades, the Peruvian Amazon has experienced ecological and social transformation with intense agriculture, petroleum extraction, road construction, and deforestation ^22– 24^. More intense and frequent climatic events represent an emergent challenge for biodiversity and, thus, for the nutrition and health of Indigenous people ^25– 27^. Increased access to communities through better roads and transportation could potentially increase access to purchasing other types of foods; however, Indigenous people reported limited access to paid jobs to generate income ^28^ and perceived that loss of biodiversity was more important than any positive benefit to their health, including food security ^29^. The long-lasting health consequences of poor nutritional status in the early stages of life could potentially delay cognitive, socio-emotional, and economic development in future Indigenous generations ^30, 31^. This implies that the current health gap between Indigenous and nonindigenous people in terms of nutritional status is likely to persist or amplify.

The following report outlines a protocol for a study designed to investigate the association of FBD on the prevalence of malnutrition-related anaemia and assess the role of FBD on resilience to extreme flooding events. This study will contribute to filling the gap needed to develop policies that address malnutrition among Indigenous people in the Amazon region ( https://www.mef.gob.pe/contenidos/archivos-descarga/anexo_DS068_2018PCM.pdf). It aligns with the Peruvian plan for sustainable development ( https://www.gob.pe/institucion/ceplan/informes-publicaciones/867912-peru-informe-nacional-2018-para-el-desarrollo-sostenible) that highlights the importance of protecting biodiversity while adapting to climate change ( https://www.ceplan.gob.pe/visionperu2050/).

## Study protocol

### Study site and context

**Figure 1.**
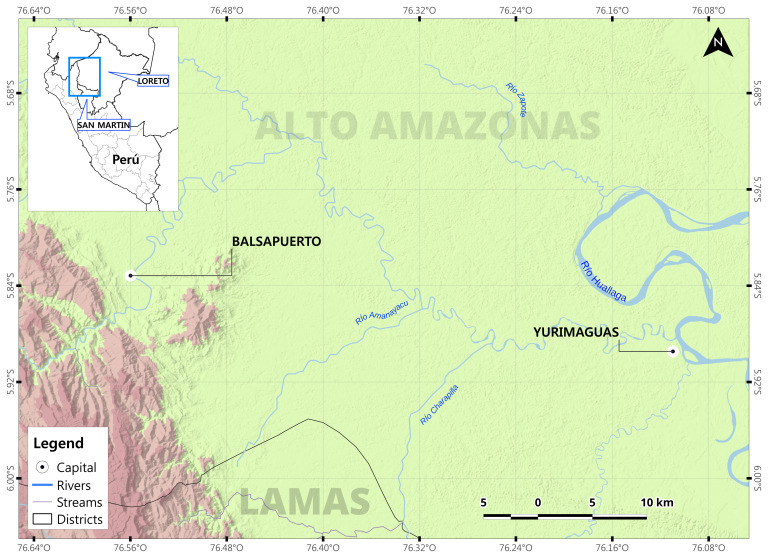
Map of Balsapuerto district, Loreto Region location.

This investigation will be conducted in partnership with Shawi Indigenous peoples from the Peruvian Amazon Rainforest. Over 25,000 Shawi people are distributed within 185 communities in the Loreto and San Martin regions. This study will be conducted in the Balsapuerto district of the Loreto region ( Figure 1, Picture 1), in the Eastern slopes of the Andes mountains, where the majority (48%) of Shawi communities are located ^32^.

**Picture 1.**
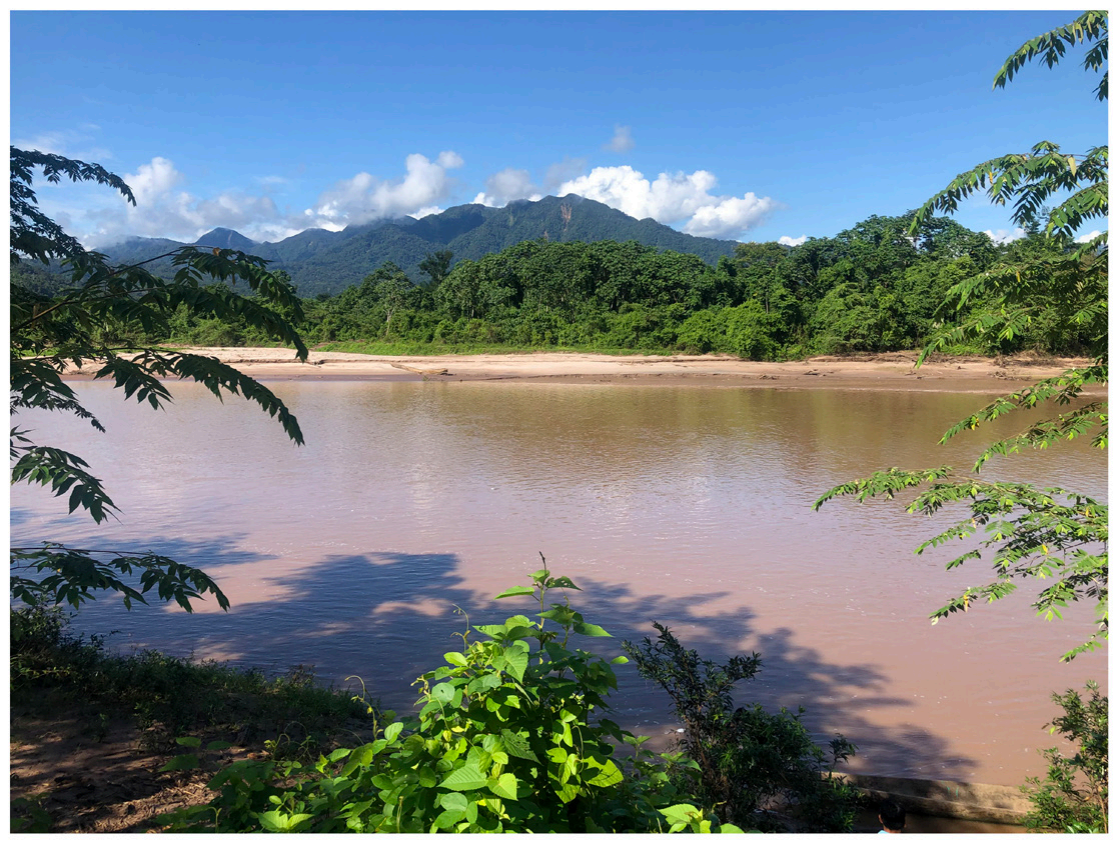
This protocol is being implemented in the Balsapuerto district of the Loreto region, in Eastern slopes of the Andes mountains in North Peru. *This picture has been reproduced with permission from Carol Zavaleta-Cortijo, May 2022.*

Shawi communities in Balsapuerto are surrounded by the Amazon rainforest and have between 50 to 100 households and a central zone typically equipped with a school and community house. Each community has a primary water source, such as a spring, creek, or stream, that flows down to the main river called *Paranapura*, which goes to the *Huallaga* river, and then to the *Amazon* river ^33^. These bodies of water are typically the primary source of water consumption for families, as there is no potable water among these communities ^32^. Shawi territory is a heterogeneous land in terms of altitude. Some communities are located at a relatively high altitude (approximately 100 meters above sea level) compared with the floodplain Amazon, while others are at sea level. This heterogeneous landscape is also observed within communities, allowing families to build houses and farms at different altitudes.

Shawi families have their main house located close to the central zone of the community, while their smaller farms are located within the range of ten minutes to one hour walking distance from the central zone. Farms usually grow key crops like cassava, maize, and plantain. Shawi families also raise domestic animals, chickens, pigs, and ducks, generally in their backyards or close to their houses. Some families also build a small, simple house made of wood and leaves in the forest, called *“Tanpu*” in the Shawi language, used for short-term overnight stays when they hunt, fish, or gather ^33^. The *Tanpu* is usually located over two hours walking distance from the central part of the community.

Regarding climatic weather in the Amazon, two main seasons are observed, according to the intensity of precipitations: wet season and dry season ^34^. Rain is constant throughout the year however, precipitation increases in intensity between October and April (wet season) and decreases between May and September (dry season) ^33^. Shawi communities do not typically experience severe floodings during the wet seasons, however, over the past five years, extreme flooding events have been more frequently reported compared to previous years. Between 1990 and 2013, only one major flooding event was recorded by Shawi participants in a previous study (1995), which is less frequent than the four major flooding events registered in the same region by the Peruvian National Institute of Civil Defense (INDECI in Spanish) over the last years 2015, 2016, 2017 and 2018. The importance of these recent changes to local weather characteristics is manifold. First, Shawi households and farms close to a body of water could be exposed to and affected by unexpected flooding events. Second, these inundations could negatively affect the availability of crops and wild foods from the forest. For this study, we plan to include communities affected by an extreme flooding event over the past five years and individuals who live in both affected and unaffected households.

### Study aim and objectives

This study aims to investigate the role of FBD on the nutritional resilience to extreme flooding events of Shawi Amazon Indigenous adults located in Peruvian communities that have experienced extreme floods in the past five years.

The research objectives are:

1.   To study the association between FBD and anaemia prevalence in Shawi adults between the ages of 15 to 60 years old (quantitative study).

2.   To investigate the role of FBD on the individual, household, or community responses to extreme floods in the past five years through a community-based participatory research (CBPR) study (qualitative study).

## Methods

### Research approach

**Figure 2.**
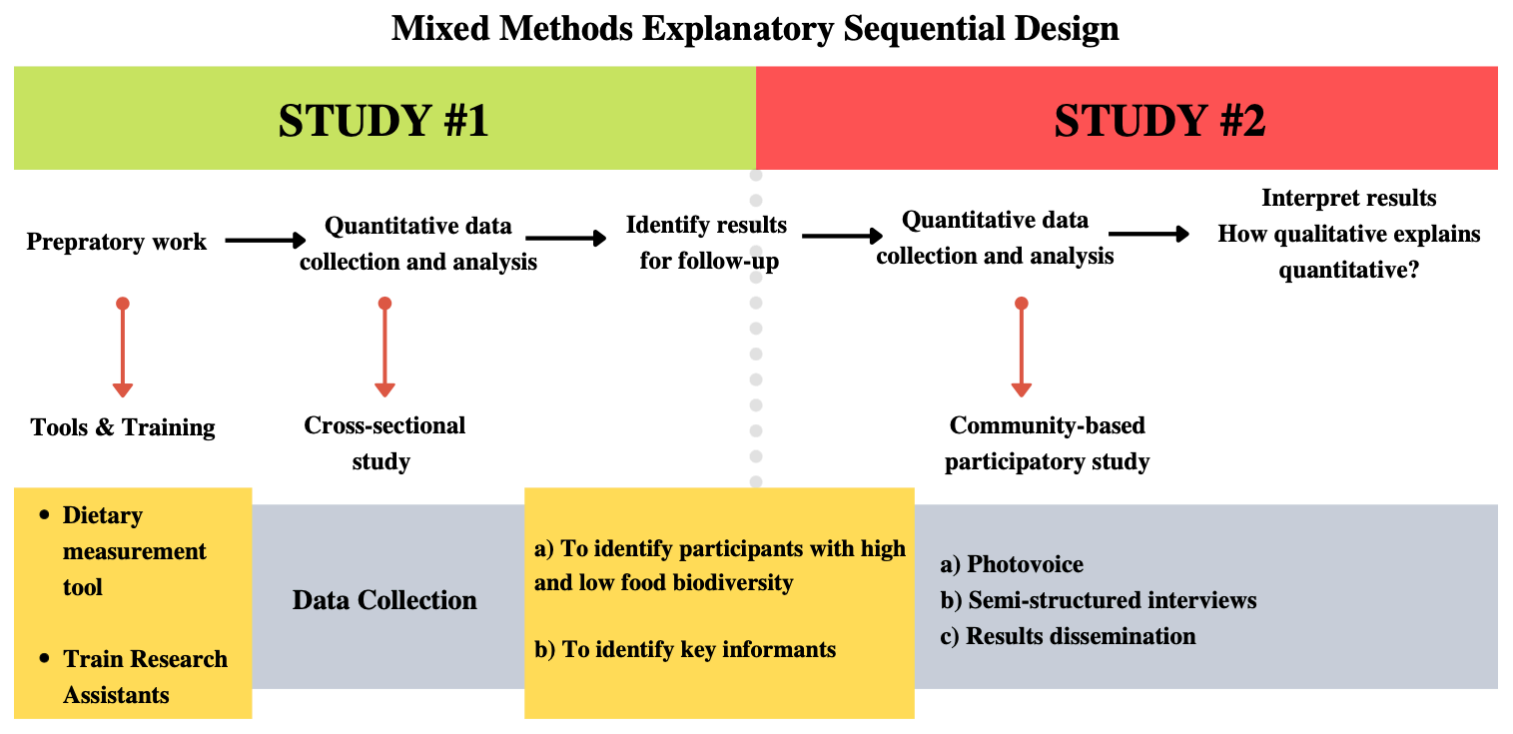
Mixed methods explanatory sequential design approach is described above by key activities per each method.

We will use a mixed methods sequential explanatory study design ^35, 36^. Using quantitative and qualitative methods will provide a better *comprehension* of the role of FBD in nutritional resilience to extreme flooding, *facilitate* the identification of people using FBD, and ensure the *voice* of Shawi collaborators will be taken into account within this study ^37^. The cross-sectional study will investigate the association between FBD and anaemia prevalence. We expect that this quantitative study will assist in clarifying whether people with higher levels of FBD will have a lower prevalence of anaemia than those with lower levels of FBD. This association may represent a potential mechanism of connection between FBD and nutritional resilience through better access to a variety of nutrients. In addition, individuals with high and low FBD will be invited to participate in the qualitative study to better understand the use of FBD, and its role in nutritional resilience to past extreme flooding events. The qualitative study will follow a community-based participatory approach by using Photovoice and semi-structured interviews to investigate the role of FBD at the individual, household, and community levels. Data from the two studies will be integrated and used to identify resilience indicators. Figure 2 illustrates the mixed methods approach and sequence of data collection. Results of the quantitative study will identify whether FBD is associated with anaemia, and the qualitative study will identify participants with different levels of FBD. Prior to the cross-sectional study, a pilot phase will take place with our Shawi key collaborators (MPT, RPI, GLR and JCHH). This pilot phase will include updating the list of previously collected FBD species included in the Extended data (1) ^38^ and hosting a cooking workshop to identify common food preparations for the diet evaluation. Pilot phase activities will allow to create a visual aid (Photo food book), to be used posteriorly during data collection.

### Theoretical framework

A climate change resilience approach to investigating household food systems with a focus on FBD and human nutrition represents an opportunity to identify responses, mechanisms, and sustainable risk reduction strategies “to provide the right food at the right time for each individual” ( https://www.fao.org/3/i3777e/i3777e.pdf), while simultaneously informing efforts to adapt to climate change ^39^. Food system resilience builds upon concepts of socio-ecological systems that consider food security as a main outcome ^40– 42^. Food system resilience has been defined as the “capacity over time of a food system and its units at multiple levels, to provide sufficient, appropriate and accessible food to all, in the face of various and even unforeseen disturbances” ^43, 44^.

The resilience framework for food and nutritional security recognizes the ability of individuals, households, communities or the whole system to cope, adapt, and transform when facing a shock or stress ^45^. In parallel to these three dimensions of resilience, it is important to identify the presence of key drivers of socioenvironmental vulnerability and to understand its relevance to the food resilience of Indigenous people. Drivers of vulnerability have been reported among Indigenous people in the Amazon ^28^, and without tackling them, efforts to adapt to climate change could be undermined. Some of these vulnerabilities have their roots in the colonization process that Indigenous people experience and include: land dispossession, constraints to the use of Indigenous languages, and limited control over changes in their territories and livelihoods ^46^.

***Food resilience in this study.*** In this study, the coping dimension refers to the ability to absorb the shock from events such as extreme floods and persist in producing or collecting food after the shock ^41^. Examples of coping may include accessing food from family, reducing the number of meals per day, purchasing locally grown foods, or being able to receive food aid. The adaptive capacity dimension implies long-term changes based on an ongoing learning process and investing economic resources to respond to present or past shocks ^47^. For example, this includes identifying and producing foods resistant to flooding based on knowledge or experiences with past weather events. Transformation dimensions represent the capacity to create a whole new system, given that the adaptation is no longer sufficient to deal with the shock in the present ^48, 49^. While coping and adaptive capacity dimensions represent the process of protecting original food systems, transformation implies a significant change in the whole system. The transformation could include fundamental changes altering social, cultural, and behavioural norms to protect their food and nutritional security in the present and the future. For example, this could include changing their food preferences and consumption from growing food in their lands or gathering from the forest to purchasing food in the city because more extreme flooding events are decreasing their access to food biodiversity. These three resilience dimensions are considered human responses to shocks, and Shawi people could implement them at different levels: individual, household or community. In parallel, we will identify potential drivers of vulnerability that could undermine Shawi responses to climate change. For example, access to health services during a flooding event is not always available in Shawi language. 

## Quantitative study

### Research questions

1.   Is higher FBD negatively associated with anaemia prevalence?

2.   Is the association between FBD and anaemia prevalence mediated by the type of FBD consumed?

### Study design

Cross sectional study.

### Participants

Our partners from the *Red de Salud Alto Amazonas*, the *Gobierno Territorial Autónomo de la Nación Shawi* and Shawi investigators (GLR, MPT) will contribute information on approaching the communities located in the Balsapuerto district. We will conduct a preliminary visit to each community to consult with their authorities about their experience of extreme flooding events over the past five years and to explore their willingness to participate in the study.

***Inclusion criteria.*** All Shawi adults between 15 and 60 years of age that live in a community impacted by at least one extreme flood since 2017 will be eligible to participate. Two participants (male and female) randomly selected within each household will be invited to participate. Individuals that declare to have lived in the community for at least one year from the visit will be included. Due to different land altitudes, not all individuals or households within a community are directly affected by floods, implying that both participants that have been directly (individual or household) and indirectly (community) exposed to floods will be included.

***Exclusion criteria.*** Shawi adults who live in communities unaffected by an extreme flood since 2017 and people who declared to have lived in the community for less than one year from the visit. We are excluding participants over the age of 60 years old since anaemia could likely be associated with multiple chronic health conditions ^50^ and less likely with FBD.

### Study variables

***Outcome.*** The primary outcome will be anaemia (yes/no). Anaemia will be determined using the WHO haemoglobin concentration cut-off points for 15 years of age and above: <12 g/dL for non-pregnant women, <11 g/dL for pregnant women, and <13 g/dL for men ( https://apps.who.int/iris/bitstream/handle/10665/85839/WHO_NMH_NHD_MNM_11.1_eng.pdf). For women at post-partum period (the period from childbirth to six weeks after delivery) anemia will be when haemoglobin is <11 g/dL ^51, 52^.

***Exposure.*** FBD will be categorized into high and low FBD. FBD is defined as all edible animal and plant species collected from the forest and/or grown on Shawi farms based on all species available in households over a one-month recall period. Participants with high FBD consumed at least 10 different species, and participants with low FBD consumed less than 10 different species in the last month prior to the interview. It was reported that 10 species was the average number of food species consumed by adult women in the tropics within one year ^12, 14^. Species included different types of birds, fish, big and small animals from the forest, insects, fruits, legumes, and palms outlined in Extended data (1) ^38^.

***Confounder.*** Extreme flooding events could create conditions conducive to other infectious diseases (e.g. malaria mosquito vector) ^53^ that could affect the prevalence of anaemia while simultaneously interrupting crop production, availability of forest foods, and decreased FBD. We anticipate adjusting for inundations. We will also explore whether flood has a role as a potential effect modifier of the association of FBD and anaemia. Exposure to flooding will be structured as participants living in a Shawi household that has experienced at least one extreme flood in the past five years vs participants located in a Shawi household that did not experience floods directly in the past five years. Based on our previous investigation with Shawi communities, floods are considered extreme when water submerges farm fields, damages crops and/or housing, and/or injures or kills domestic animals ^28^. Age, sex, and other socioeconomic factors would be considered confounders.

***Mediator.*** It is known that non-haem iron (from plant-based food) is not as well absorbed as haem iron (from animal-based food) ( https://apps.who.int/iris/handle/10665/42716), implying that consumption of different food groups could also be associated with anaemia prevalence. Therefore, to answer the second research question, we will create a variable called “type of FBD consumed” with two categories based on the most predominant type of food species consumed: FBD from animal food species and FBD from plant food species. Food species will be grouped based on recommended guidelines ^54^ to measure household dietary diversity. Animal food species group will include meat, eggs, and fish. Plant food species group will include cereals, white tubers and roots, vegetables, fruits, and legumes. Participants that reported consuming a higher number of animal food species compared with the number of plant food species, will be considered as having their type of FBD from animal food species, whereas when the number of plant food species exceed the number of animal species consumed, will be considered as obtaining their FBD from plant food species. However, since there is not yet an accepted measured for consumption of food biodiversity ^55^, we will be analyzing the data to explore whether is possible to create another indicator to better reflect what participants eat.

One key pathway of association between FBD and anaemia includes dietary iron intake. We anticipate estimating the participants’ absolute (mg/d) total iron intake haem and non-haem. Dietary iron intake will be described by the distribution’s median, 5 ^th^, and 95 ^th^ percentile of the distribution. For this study, we will use ≥50 ^th^ and <50 ^th^ percentile to determine high and low iron intake levels for women, men, and pregnant women. Iron content in food will be calculated using information from the Peruvian food compositional table ^56^ and complemented with the Brazilian food compositional table ^57^, Centro American food compositional table ^58^, and Awuajun Indigenous food table ^59^.

### Sample size

We anticipate that a total of 350 Shawi female and male adults between 15 and 60 years old will be invited to participate in this study. A previous survey conducted with Shawi adults in 2015 indicates that prevalence of anaemia is around 35% ^60^, that mean Hb (SD) concentration is around 12 (1.5) g/dL, with intrafamily correlation around 0.1. With an anticipated 40% of participants in the exposed group (high FBD) and 60% participants in the unexposed group (low FBD), we will have 90% power (at p<0.05) to detect a 0.4 g/L difference in Hb concentrations, and more than 80% power to detect a 16 percentage points difference in absolute terms in the prevalence of anaemia (e.g., 27% vs 43%)

***Adjustment to sample size for COVID-19 restrictions.*** We are also preparing a case scenario where we can not reach our sample size because COVID-19 restrictions or COVID-19 transmission is still a risk for participants in the field. A smaller sample size of 280 participants, rather than 350, will have 88% power to detect a 0.4 g/dL difference in Hb concentration, and in terms of anaemia, 80% of power to detect a 17 percentage points difference instead of the 16 percentage points difference we had originally powered to detect. 

### Procedures

The study will begin with a pilot study. During the pilot phase, a cooking session will be performed to create a food photo book (FPB) to show the portion sizes of typical foods and Shawi preparations. We will follow the experience of working with rural communities in Latin America ^61^ to create a Shawi FPB. During this activity, participants will be asked to take us to their farms to record information about edible plant and animal food species to be included in the diet evaluation in the cross-sectional study. 

After the pilot, the following procedures will be performed on each participant:

***Interview.*** An adapted version of a previous questionnaire used to investigate Indigenous health and adaptation to climate change will be used ( https://www.ihacc.ca/). The questionnaire includes 1) individual sociodemographic information: age, sex, education, paid work, productive food activities over the past two weeks (planting, harvesting, weeding, fishing, hunting, collecting food in the forest), income, pregnancy status, 2) health status: previous diagnosis of any chronic health condition, fever over the past two weeks, presence of diarrhea, and consumption of any medication or traditional medicine; and 3) household characteristics: access to government food or social programs, history of flooding over the past five years, and primary source of water and sanitation.

***Diet evaluation.*** A food frequency questionnaire (FFQ) with a four-month recall period will be applied using an updated version of a list of foods rich in iron in the Extended data (1) ^38^. A 24h-dietary recall (24HR) will be collected from each participant. We will use the food photo book created in the pilot study as a visual aid and plan to bring a weight scale in the case that a participant is willing to show us the amount of food consumed. We plan to use an offline version of the digital tool *myfood24* for the dietary recalls ^62^. The utilization of this tool will allow for a more efficient dietary assessment by collecting food information systematically and automatically generating detailed food and nutrient intake results.

***Anaemia evaluation.*** Anaemia will be evaluated using two methods:

1.   Hemoglobin: Hemoglobin (Hb) concentration will be measured using one finger prick of blood (approximately 10 μL) with a hemocue.

2.   Serum Ferritin: For those who test positive for anaemia with the hemocue, a sample of serum (2ml) will be taken to determine whether the anaemia is related to an iron deficiency. A quantitative C-Reactive Protein (CRP) measurement will be taken on the sample. The CRP measurement will assist in identifying whether the value of ferritin could have been altered by any inflammation or infection ( https://www.who.int/publications/i/item/9789240000124). Ferritin levels could be raised in populations where inflammation or infection is likely present (e.g., blood parasites or chronic diseases). The measurement of CRP is recommended to adjust ferritin cut-off levels to determine iron deficiency. To define iron deficiency anaemia, we will use a cut-off level for serum ferritin of <15 ug/L when CRP is <5mg/L and serum ferritin level of <70 ug/L when participants have a CRP ≥5mg/L ( https://apps.who.int/iris/handle/10665/337666). Serum samples for CRP and ferritin measurements will be sent to a laboratory in the closest city, where a fluorescence immunoassay will be performed.

***Health evaluation.*** This evaluation will allow us to detect other health conditions that may be associated with anaemia from non-dietary causes such as chronic inflammation or infectious diseases. Health evaluation will include measurements of body temperature, blood pressure, malaria testing, anthropometry, and intestinal parasites. 

***Body temperature.*** A digital thermometer will be placed in the armpit to measure body in centigrade and to identify potential malaria cases.

***Blood pressure.*** A validated automatic blood pressure monitor will be used to register participants’ blood pressure. Participants will be invited to sit and rest for at least 10 minutes before blood pressure will be taken. We will conduct only one measurement. 

***Malaria test.*** A blood smear will be taken to test for malaria on those who reported having had a fever within the past two weeks. This analysis will be performed at the health center to the Ministry of Health in the Balsapuerto district.

***Anthropometry.*** Weight and height measurements will be used to calculate body mass index (BMI).

***Stool samples for parasite evaluation.*** Stool samples in 5% formalin PBS will be evaluated using direct smears and the standard Formalin-ethyl acetate concentration method. We are interested in identifying hookworms and their association with anaemia presence ^63^. The Infectious Diseases Research Laboratory of the LID, at UPCH will be responsible for processing the stool samples.

### Data collection

**Figure 3.**
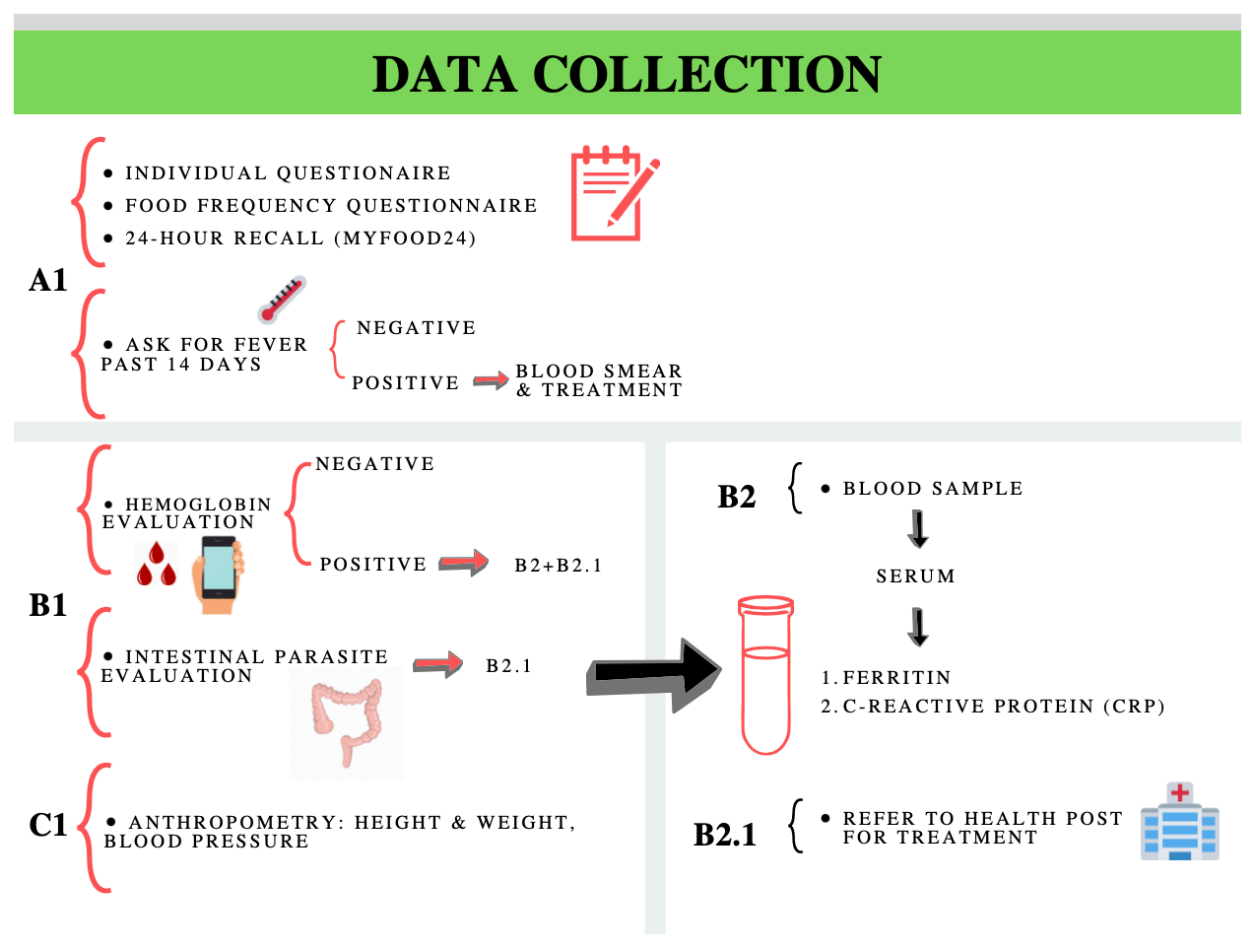
Sequence of how diet and health evaluation procedures will be applied during data collection.

Two research teams will be organized: 1) Food interviewers (FI) led by the PI (CZ, RS, and GL) and 2) Health evaluators (HE) led by a senior biologist (CFN) with extensive experience working with the Shawi population including performing venipuncture in the field. Shawi research assistants (MPT, JCHH, and NIT) will be part of the interpretation team and assist with interview and results communication. Each participant will be visited at their home, and after providing written consent, the interview, 24HR, and FFQ will be completed. After the interview, the HE will visit the participant to record their weight, height, blood pressure, and hemoglobin concentration with the hemocue. All participants will be given a small container to self-collect a stool sample. A research team member will collect the stool sample on the following day. For those with anaemia identified by the hemocue, 4 mL of blood will be extracted to separate the serum for the ferritin and CRP measurements. The malaria test will be performed at the closest health center in Balsapuerto. The serum sample of those diagnosed with anaemia will be refrigerated and sent to a local laboratory to analyze ferritin and CRP. The stool sample will be sent to UPCH. Figure 3 shows the sequence of procedures to be applied during data collection.

### Statistical analysis

Statistical analysis will be conduct using Stata version 17. Descriptive analysis will characterize the populations’ socio-demographic characteristics and food habits of the population. Fisher´s exact test will be used to assess the association between FBD (low/high) and anaemia (yes/no). Generalized linear models and prevalence ratios (PR) will be used to investigate the role of FBD on anaemia, adjusting for exposure to extreme flooding in the past five years. We will explore the role of flooding as a potential effect modifier. As a secondary analysis, we will explore the causal route between FBD and anaemia based on the different types of FBD consumed (FBD from animal food species and FBD from plant food species) and dietary iron intake. Non-independence of observations (participants within the same households, or possibly within the same communities) has been allowed for in estimating the sample size requirements and will be taken into account in statistical modelling through use of Sandwich estimators where necessary.

**Figure 4.**
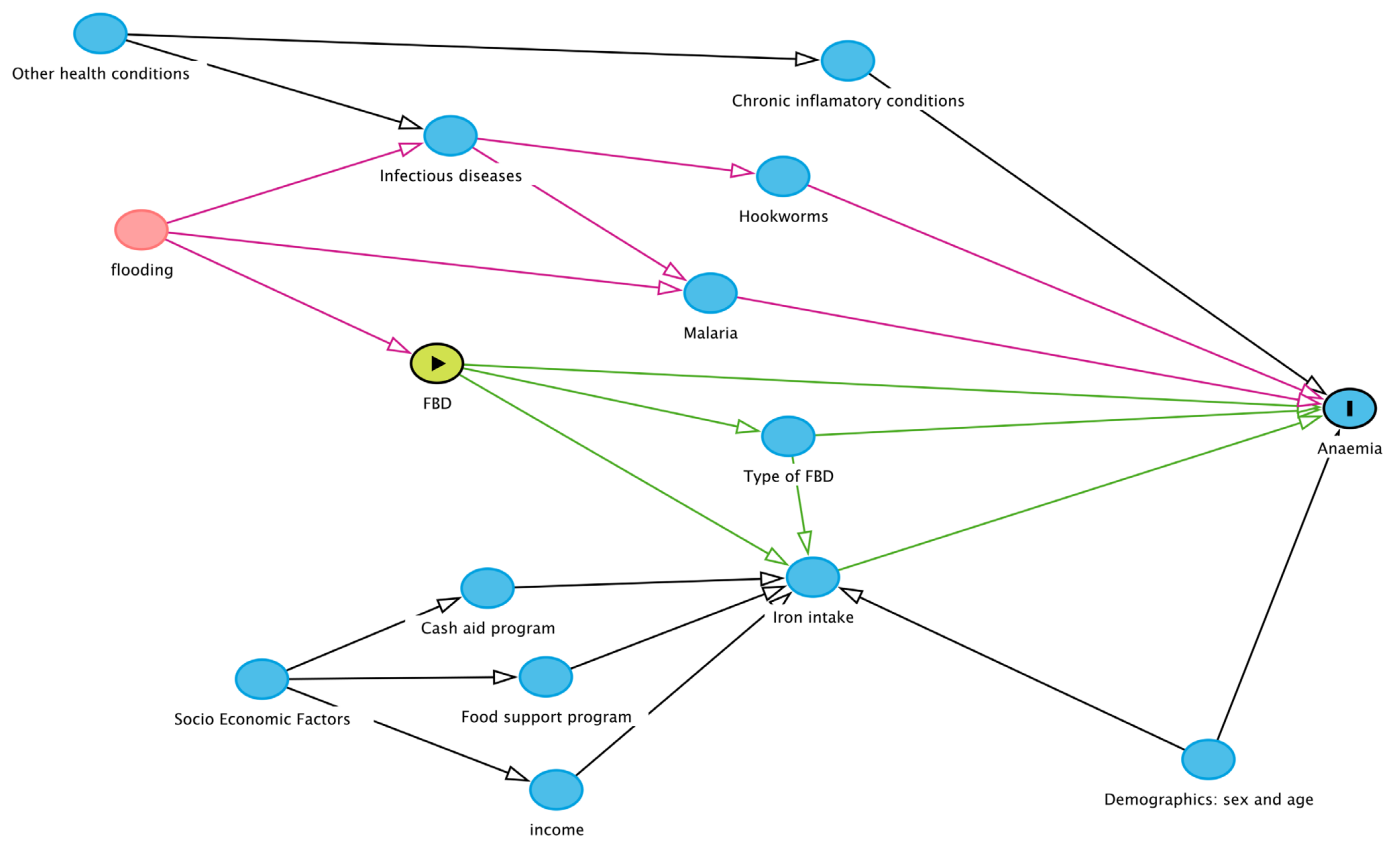
DAGitty figure displaying the causal diagram assumption for the quantitative study. It displays the causal diagram assumption for the quantitative study. Primary outcome (anaemia prevalence), primary exposure (FBD: food biodiversity), potential mediator (type of FBD and iron intake), and potential confounder or effect modifier (flooding). The figure also illustrates other covariables to be analyzed, including demographic (sex, age), socioeconomics (cash aid program, food support program and income), and other health conditions (infectious and chronic diseases).

Figure 4 illustrates the assumption for the casual model using the DAGitty graphical tool ^64^.

## Qualitative study

The qualitative study seeks to answer the following research question: Does household food biodiversity favour the resilience of Shawi household food systems to extreme flooding events?

### Study design

Community-based participatory research (CBPR). This design promotes the involvement of the participants as collaborators within each phase of the study. This is particularly relevant for the Indigenous context, given the exclusion and invisibility of their culture, livelihoods, and medical practices, which have disadvantaged them from receiving practical benefits from food and health interventions. CBPR follows key principles for partnership, including ^65^: projects are community-driven, projects promote co-learning, the dissemination of results are in a useful term, there is a guarantee that the research is culturally appropriate, and the project recognizes Indigenous food sovereignty. This study protocol builds upon previous work with Shawi communities that identified food and nutritional security as a priority for Indigenous households, community members and local authorities. We are partnering with Shawi Indigenous local and national leaders who are part of and co-authors of this investigation.

### Sample size

Twenty-eight adults (14 male /14 female) from a previous study with high and low FBD will be invited to be collaborators in a Photovoice group activity. Semi-structured interviews will be conducted with key informants, female and male adults, to explore the impacts of past extreme flooding events on FBD. We anticipate interviewing 10 participants however, data collection will cease once the saturation is achieved.

### Procedures

***Photovoice.*** Photovoice has been recognized as a method to encourage participants to share their voices through pictures and to choose what to share, why it was taken, and what it means to them ^66^. Our research group has used photovoice to characterize Shawi food security with good performance and acceptability among adult participants ^28^.

**Figure 5.**
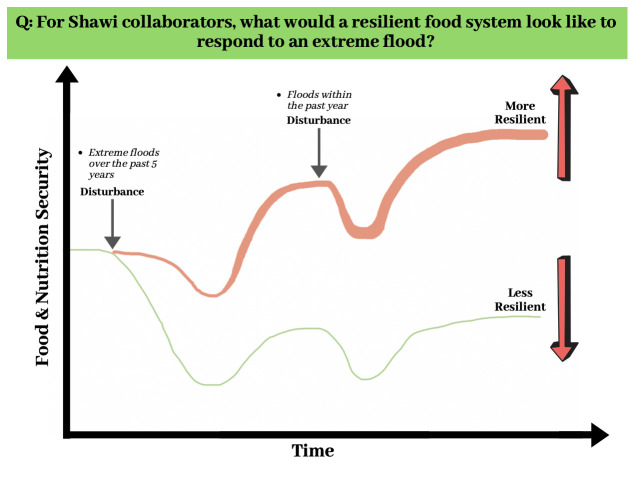
The resilience of Shawi food and nutritional security to extreme flooding over time. We plan to explore and validate this figure with collaborators in the photovoice.

The Photovoice activity will include three workshops with Shawi collaborators: 1) to refine the research question and provide instruction, 2) to identify key pictures and elaborate on the narrative of FBD and food system resilience, and 3) to present and discuss the main findings. Digital cameras will be provided to each participant to help them to illustrate their answers. Examples of potential questions include: What would a resilient food system look like in response to an extreme flood? What would be the role of FBD in promoting the nutrition of Shawi people before, after, and during an extreme flood? What factors will facilitate the use of FBD to promote a resilient food system? We will use the information in Figure 5 to explain the initial research concept of resilience to extreme flooding events to our Shawi collaborators. During the initial meeting, our collaborators will be instructed on the ethical aspects of including faces in their pictures and discuss which policymakers they would like to transmit their results to. A final result dissemination meeting will be planned collectively with the collaborators. During the dissemination meeting, other members of the community and local authorities may be invited to attend to motivate action that benefits the Shawi nutritional status in facing extreme flooding events. 

***Semi-structured interviews.*** Results from the photovoice activity will be used to develop a guide for the semi-structured interviews that will explore nutritional resilience and the impact of extreme flooding on FBD. We anticipate that the guide will include information to complement the three key themes of the theoretical resilience framework: coping, adapting, and transforming. Semi-structured interviews assist in collecting additional data, especially if specific households or Shawi collaborators report experiencing a new extreme flooding event. The Shawi research coordinator (GL) will stay in the district for the entire duration of fieldwork (12 months) to monitor each community and provide monthly communication with the PI and the collaborators at the Red de Salud Alto Amazonas.

***Shawi collaborators as researchers.*** This protocol was designed and will be implemented in close collaboration with Shawi community members listed as co-authors: GL-R, MP-T, RP-I, JCh-H & NI-T. Members of our research team are young and adult Indigenous people from Shawi communities that are participating in this study. Shawi collaborators were invited to participate in this study for the following reasons: GL-R is a health technician, Shawi researcher who has been working with the PI (CZC) in previous studies on climate change and food security. GL-R is also the first Shawi people recognised by the Peruvian cultural ministry as an official Indigenous translator among their people. MP-T and RP-I are husband and wife, community members and Shawi leaders selected by their own community to be part of this study. JCh-H & NI-T are Shawi youth nurse students who were recruited based on their advanced knowledge of Shawi and Spanish language, especially writing skills. Shawi researchers have provided this protocol with crucial information on the definition and Shawi conceptualisation of floods and other weather characteristics in their communities. A version in Shawi and Spanish key terminology is attached see Extended data (2) ^67^. During the fieldwork, we plan to validate this concept list with the participants to have a better understanding of how Shawi collaborators understand extreme climatic events. In addition, these Shawi researchers will provide constant advice to navigate the differences between Spanish and Shawi languages, on culturally acceptable ways of completing community consultation, and guide us through the Indigenous land to reach communities and households.

***Revision of key literature.*** Given the multiplicity of disciplines working on biodiversity, culture and adaptive social strategies to responses to environmental changes in the Amazon region ^68, 69^ and among other Indigenous people ^70, 71^, we are going to use previous ethnographic work to inform our Photo Voice workshop and Semi-structured interviews. We plan to include a social family and community networks dimension in order to make sense of the long-time relationship among forests, foods and Indigenous people ^72, 73^, with a primary focus on identifying key resilience strategies to protect humans of getting anaemia.

### Analysis

The Photovoice analysis will be done with our Shawi collaborators mainly during the third workshop by identifying challenges and opportunities related to food biodiversity and their nutrition in response to extreme flooding events. The results will be incorporated into the resilience framework, emphasizing concepts and understandings that our collaborators will share with us.

Semi-structured interviews will be analyzed following the three dimensions of the resilience framework analysis: coping, adaptation, and transformation. After coding the data, manifest and latent analysis of the content will be completed, and how respondents refer to specific topics or describe their experiences with FBD and flooding events will be examined. The information will be transcribed, incorporated, and analyzed with NVivo software version 12. The analysis will be iterative, and during a follow-up visit, emergent themes will be presented to the participants and validated by them.

### Integration of quantitative and qualitative data

We will use a mixed methods sequential explanatory study design. Our previous research has found that multiple non-climatic stressors represented major determinants that increase vulnerability of Shawi food security to climate change, including increasing population, natural resource degradation, and lack of opportunities to increase local income ^28^. Similarly, Shawi have reported important elements in their food systems that could increase opportunities to adapt to climate change, for example, Indigenous ecological knowledge, sharing food networks, and food preparations based on local resources ^28^. To better understand the role of these Indigenous responses and inform the design and monitoring of food and nutritional policies, this mixed method study aims to inform the development of key indicators of resilience for coping, adapting, and transforming to protect human nutrition.

We anticipate that indicators would include data collected from the quantitative study (e.g., anaemia status, biodiversity of food in diet, income, access to social support programs) and will be complemented by investigating other key characteristics for each theme. For example, Indigenous knowledge about edible wild species is key to enhancing dietary diversity ^13^, including during extreme climatic events ^74^. Practices of traditional food preparations such as fermented foods provide safe and culturally accepted edible options to community members within the Shawi and among other populations ^75, 76^. Given that anaemia is also a result of other health conditions like intestinal parasites, chronic inflammation, and excessive bleeding related to pregnancy, adequate access to health care services may decrease the probability of having anaemia while simultaneously increasing the adaptive capacity of our participants to respond to climatic risks that threaten their health ^77^. Financial services are incorporated as a proxy for having a job or access to social support programs given the potentiality that economic resources have on enhancing diet diversity with non-Shawi typical food and/or by the condition to complete regular health checkups to receive the benefit ^78, 79^. The transformation dimension will include changes in cultural, social, or economic characteristics, for example, accessing and accepting new types of nutritive foods. Women are key actors in supporting nutrition and food security and the use and preservation of biodiversity among Indigenous communities ^80^. However, some cultural traditions constrain women’s autonomy in taking on leadership roles regarding food and nutrition, globally ^81^, including within some Indigenous populations ^82^. We seek to understand to what extent women participation can transform the Shawi Indigenous food systems to protect nutrition and foster resilience, especially during extreme climatic events.

**Figure 6.**
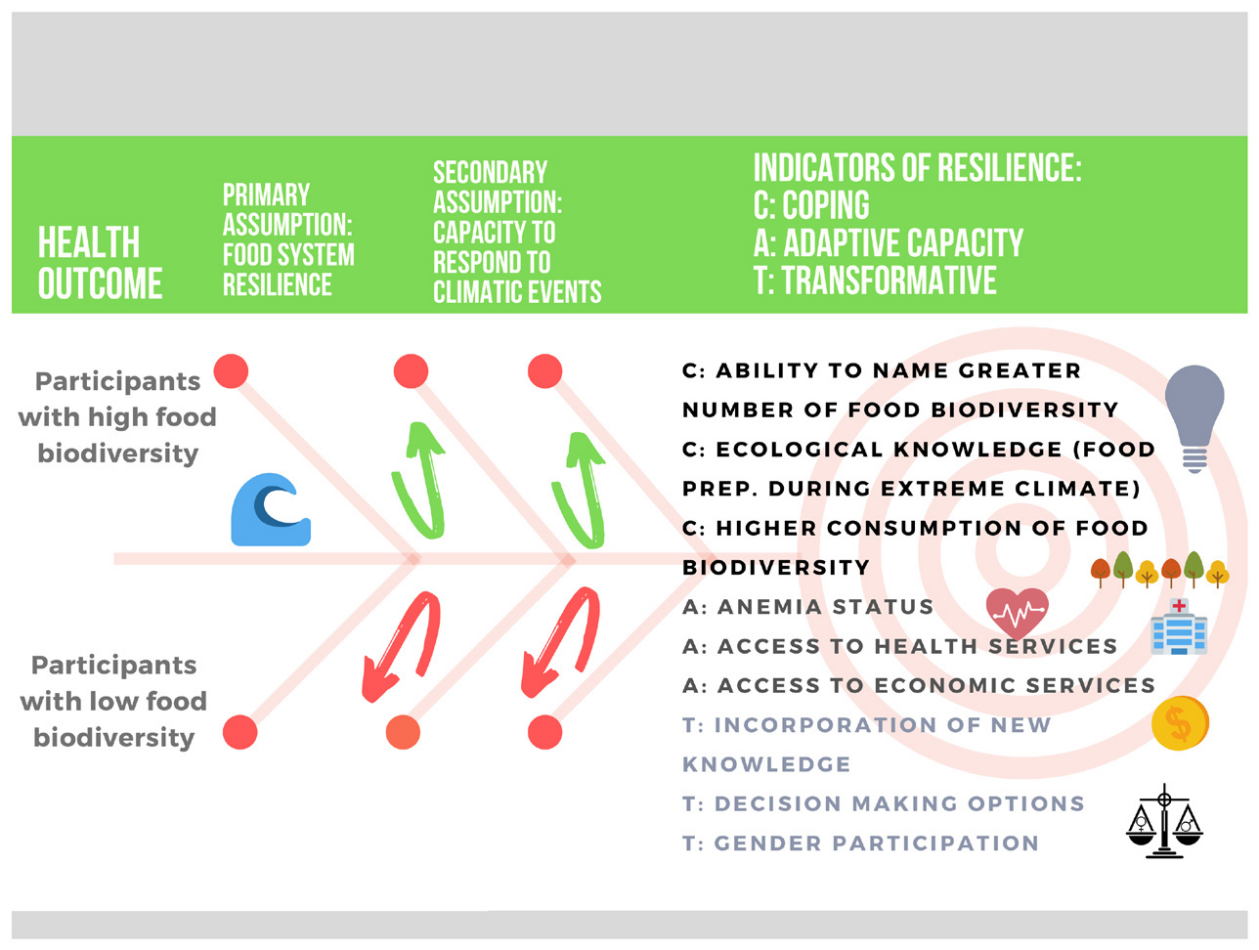
Potential resilience indicators to be explored and validated with Shawi participants. The proposed indicators are based on the work of previous researchers and are grouped in three main dimensions: coping, adaptive capacity, and transformation.

Figure 6 illustrates our assumption for main themes (coping, adaptive capacity, and transformation) and key indicators to be explored along with others that may emerge during the data collection. Even though we propose these indicators, our approach will be flexible to allow the validation, rejection, and/or emergence of new indicators relevant to our Shawi collaborators. Since nutritional status is typically an indicator at the individual level, we will use the resilience framework to include information from the qualitative study and develop indicators at the individual, household and/or community level, according to the responses of our participants.

## Ethical considerations

All data collection will be facilitated by Indigenous trained research assistants. This study has been approved by the Universidad Peruana Cayetano Heredia Institutional Ethics Board SIDISI:104343. All participants will provide a written consent form.

### Community relationships

This study builds on over ten years of partnerships with Shawi communities and local researchers who have previously identified food security and nutrition as key climate health outcomes ^29^. Interest in understanding the Shawi diet resulted from the previous conversation with Shawi participants who desired to keep their Indigenous food systems alive and continue using their local food and biodiversity as the main source of their diet ^78^. Over these past years, we have built relationships based on mutual trust and respect with Shawi authorities and Indigenous leaders at a local and national level. GL will be employed as a local coordinator of this study and will be the main liaison with the Shawi communities. We also plan that one community benefit will be a workshop about Indigenous food and nutrition led by Indigenous women from the organization AIDESEP. This workshop will be funded by the study and allow these women to travel to the communities and share their Indigenous knowledge about local food species and food preparation with our Shawi participants.

### Community consultation

Prior to the fieldwork, a community assembly will be organized to explain the research objectives and methodologies and invite adult volunteers to participate. Based on our previous experience, the community assembly is the best Shawi Indigenous organization to perform a proper cultural consent process and inform potential participants ^83^.

### Confidentiality

All the participants will be allocated a unique code. The names will be kept in a separate sheet/file associated with the identifier codes. The questionnaire and digital information will only register the codes of participants. Biological samples will be processed with the codes only. The final databases will be analyzed with code only. All data sets will be de-identified and kept in a computer with a password dedicated to the project.

The complete qualitative study will be conducted in the local Indigenous language and translated to Spanish. Translators will be trained on keeping the confidentiality of the data collected. We will ask for participants’ consent to record the photovoice activity and the interviews. The recorded information will be transcribed to be analyzed together with the pictures that participants agree to share. We will keep the information on sex and age in the transcripts as it is important for the analysis. However, identification information in each transcript of the recorded interviews will be erased. The transcripts will be stored on a computer that can only be accessed by a code known by the principal investigator. Only the principal investigator (CZ) will have access to this database. Once the final report is completed, the recordings will be destroyed. In the case of photographs, authorization will be requested for the use of the photos during group meetings. If the selected photos contain the image of any person, the field researcher will see to locate the photographed individual and request their permission for the use of the photo in academic meetings and dissemination of results.

## Results dissemination

We will disseminate the results to the participants and provide a health and nutritional instructive session in their own language. We will ask permission from participants to communicate the results to the local health post to provide the standard MoH treatment for anaemia and parasites. Peruvian MoH protocol indicates treatment with mebendazol 500mg single dosage and iron supplements for those with iron deficiency anaemia ( https://www.gob.pe/institucion/minsa/normas-legales/189301-479-2017-minsa). These communities belong to the jurisdiction of the Red de Salud Alto Amazonas, who have agreed to collaborate and facilitate the treatment for those cases.

Indigenous national and local organizations will be informed and invited to provide comments and support the dissemination of results. A community advisor board (CAB) has been organized to assist in developing the complete research process. The CAB will be instrumental in conducted this study from both, nutritional and cultural approaches. For example, the CAB will advise the optimal way to represent the Indigenous realities and focus on key food and nutritional aspects that are relevant for the benefit of Indigenous communities. Senior nutritionists from the Centro Nacional de Alimentación y Nutrición (CENAM) and women Indigenous leaders from the Asociación Interétnica de Desarrollo de la Selva Peruana (AIDESEP) will help us to settle the CAB. We will prepare policy briefings to highlight key findings to address the attention of policymakers at local, national, and global levels.

## Discussion

The nutritional dimension is often lost in the agricultural concerns when considering the impact of climate change on food security and resilience ^84^. This mixed methods study emphasizes the nutritional dimension of Indigenous food systems, and considers that a food system is resilient when it is able to cope, adapt, and transform to protect human nutrition ^45, 84, 85^. As climate change progresses globally ^5^ and deforestation associated with commercial agriculture ^86^ and rapid urbanization ^87^ rises, there is a heightened risk of biodiversity loss in the Amazon. Consistent decline in biodiversity affects populations who base their diet directly on natural resources as they lose opportunities to use cultural, well-accepted food resources to improve their nutrition.

By targeting anaemia as the main outcome, this study will contribute to the information gap on the biological and environmental determinants of a prevalent health condition in Peru amongst one of the most vulnerable populations to climate change in the Amazon region ^88^. Investigating the role of FBD on nutritional outcomes and the pathways that connect climatic risks, diet, and nutritional status are particularly relevant for Amazonian Indigenous populations such as those in the Peruvian Amazon. Indigenous communities in the Peruvian Amazon are considered highly vulnerable to the impacts of climate change on health and food security because their current food systems are closely related to their natural surroundings ^28, 89– 91^. In a previous study, we found that biodiversity was an important element of dietary behaviour for Shawi Indigenous families. We were able to list a total of 149 types of food named by participants during an exploratory study following ten households over a 12-month period see Extended data (1) ^38^. More frequent extreme events could be threatening animal and plant species commonly used as food sources ^27, 92^. Furthermore, their vulnerability is likely exacerbated by the elevated rates of undernutrition affecting both children and adults ^93– 95^.

This study will identify Indigenous diets, food preparations, and key food resources that could be used in the future to inform interventions targeting the elimination or reduction of anaemia among the Shawi in a changing climate. In addition, this study will investigate other prevalent health conditions, i.e. intestinal parasitic infections, that could be associated with anaemia and that may also be relevant for planning climate change adaptations in Shawi communities or within other Amazon Indigenous communities exposed to similar environmental and social conditions. Finally, the mixed method approach will allow us to better understand Shawi responses to extreme floods, and the use of resilient indicators will serve to monitor climate change adaptation interventions in the future.

### Adaptations made because of the COVID-19 pandemic

Data collection for this protocol was initially planned to be conducted in October 2020. Also, our original plan included two follow-up evaluations to be able to account for seasonality. Since the weather conditions in the Amazon favor changes in precipitation over the year, with the main two wet and dry seasons affecting food production and food accessibility, a longitudinal approach would be preferred. A longitudinal approach could measure whether there is a change in the diet throughout different seasons and whether this change affects the level of anaemia or the association between FBD and anaemia. The lack of a seasonal approach, including quantitative and qualitative data collection, represents a limitation in this study. We have previous studies ^28, 76, 96^ with this same population that are helping us to understand seasonal changes that we plan to use to triangulate our results.

However, due to mobility restrictions introduced and the ongoing pandemic in Peru from March 2020 to the present (2022), our research team had to make essential adjustments to make the study viable, including 1) conducting the pilot phase with our research partners already located in Shawi communities, 2) postponing the start of the data collection until 2022, and 3) retaining the main research question but with only one period of data collection.

The pilot phase will involve minimum contact with people from outside the community. It will include the development of typical recipes and a food photo album to be used once we go to the field for the main study. This pilot will be conducted by Shawi researchers already located in their communities (MPT, RPP, JCHH, NIT) and led by qualified nutritionists (RSC, VMA) with the support of a psychologist (JVQ), who has extensive experience developing participatory workshops for Shawi people.

Changing political situation in Peru, including municipal elections coming in late 2022, in addition to the COVID-19 pandemic vaccination strategies poorly accepted by some Indigenous leaders ^97^, have created resistance to accepting foreign people within Indigenous communities. Some Indigenous families are suspicious about the presence of health workers or health people working within their communities and the risk that they may be forced to be vaccinated. Thus, we plan to stay the minimum duration between March and December 2022, at each community since we do not want to jeopardize the trust that has been built with our Shawi collaborators. Therefore, no quantitative follow-up will be conducted in this study.

In addition, working during the pandemic and post-pandemic periods requires that we take extra precautions before entering the communities to conduct data collection. We have established a health protocol for monitoring key symptoms (e.g., fever, cough, or rhinorrhea) and taking a COVID-19 test before data collection when symptoms are present. These adjustments require additional time and economic resources than previously anticipated and, therefore, will prevent committing to a follow-up data collection in different seasons.

Finally, the lack of electricity and adequate cellphone signal in some of the communities to visit requires travelling to the main city to get food and water supplies and regain communications. We have established a working protocol to be in the field for a maximum of 10 continuous days working with community research assistants (designated by indigenous local leaders), including the help of a person to cook for the team. The ten days will be followed by seven days of rest in the main city before data collection will continue. In addition, we will take between two to three days to mobilize from the main city to the communities. It is important to note that since Shawi families dedicate every day to going to their farms or to the forest for food, we will complete visits mainly before 8:00 AM or after 4 PM to ask for willingness to participate and to complete the health evaluation. We anticipate that following this fieldwork operation’s protocol will allow us completing the data collection in approximately three to four months to achieve our sample size. Following the quantitative phase, an additional three months will be required to complete the qualitative phase.

We acknowledge that the COVID-19 pandemic could impact the nutrition and food security of Shawi communities not only for the direct impacts on their current health condition but also for the preventive strategies to contain the spread of the infection, such as strict quarantine and self-isolation. We anticipate developing a complementary sub-study with some questions exploring the presence of COVID-19 symptoms in the previous six months and any Indigenous strategy taken to face the pandemic.

## Study status

Pilot phase has already completed. During the cooking workshops, we were able to revise and validate our original list of individual foods and species that families consumed. This list is informing the preparation of our 24-hour recall tool and is helping us to better understand how biodiversity is used by our Shawi collaborators. We have completed four cooking workshops to come out with two comprehensive list of species one for men and one for women. Please see attached lists Extended Data ^98^ 3 and 4. We are working on preparing a full publication about the process to create a Shawi food photo book for this investigation ^99^. During initial visits to invite communities, we were able to complete a first chronology and description of major floodings in the past and how external support from governmental institutions was provided aid. See summary in Extended Data ^100^ 5. We have completed data collection for the quantitative study in six communities with 340 participants who agreed to participate by providing their written informed consent. Since we are using paper format questionnaires, we have prepared digital questionaries using "Kobo Toolbox" https://www.kobotoolbox.org/ to enter the information. We are currently typing the data into the Kobo questionaries to complete the database. We are also revising the R24 information in the paper format before entering this information into the Myfood24 digital tool. We have yet to finalize the biodiversity analysis of our participants' diet to identify potential candidates to be invited to the qualitative study. We plan to complete the qualitative phase of this study between November and December 2022. Integration of data will be completed between January and March of 2023.

## Conclusion

This study takes a participatory approach to engage scientific and Indigenous knowledge ^101^ and co-create a resilience framework to extreme flooding events. It will use qualitative and quantitative methods to investigate anaemia, a complex and pressing global nutritional outcome affecting multiple populations in the Amazon region. Our findings will be integrated to identify nutritional resilience indicators that can inform adaptative interventions to changing climatic conditions in the Amazon and that respect Indigenous worldviews.

## Data Availability

No underlying data are associated with this article. Figshare: Extended Materials 1 for Protocol_List of Shawi foods, https://doi.org/10.6084/m9.figshare.20514363.v1 ^38^. Data are available under the terms of the Creative Commons Zero "No rights reserved" data waiver (CC0 1.0 Public domain dedication).
