# Supplementary material for: Does food biodiversity protect against malnutrition and favour the resilience to climate change-related events in Amazon Indigenous communities? A protocol for a mixed methods study
Source: Wellcome Open Res. 2023 Jun 26;7:246. Originally published 2022 Oct 4. [Version 2] doi: 10.12688/wellcomeopenres.18235.2 (PMC10924752; doi:10.12688/wellcomeopenres.18235.2)
Supplement: Peer review history

# Reviewer report: Reviewer response for version 2

**DOI:** 10.21956/wellcomeopenres.21703.r72298

**Published:** 2024-03-09

**Authors:** Chiza Kumwenda

**Affiliations:** 1 University of Zambia, Lusaka, Lusaka Province, Zambia

Specific editorial consideration:

 ---------implying a higher nutritional status, instead of higher nutritional status, the authors can indicate optimal or good nutritional status.

 Under study protocol, replace this investigation with this study...

 Also make sure the concepts are consistently presented. For example the design is a mixed exploratory study design, and then the Authors bring in cross sectional and then mention quantitative study. 

 Under coping studies, it would be more informative if examples of sustainable coping strategies are included.

 For 24 hour dietary recall, the authors are indicating it will be for everyone. However, for household level data only the person with primary responsibility on food preparation etc may be used to provide data. The Authors should explain who they will use the data from the two participants from the household.

 Put temperature between "measure and body" in the sentence pasted here-A digital thermometer will be placed in the armpit to measure body in centigrade and to identify potential malaria cases.

 Anthropometry. The Authors should provide the details of specific instruments which will be used to take measurements, include the brand for example of a weighing scale. The authors should also provide the formula for calculating BMI.

 Malaria test. Why will it be done only on those self reporting? individuals may be asymptomatic to Malaria. The Authors should justify the approach.

 The authors should justify the use of Fishers exact test and not Chi Square out lightly.

 Otherwise the authors have presented the protocol with sufficient depth.

Is the study design appropriate for the research question?

Yes

Is the rationale for, and objectives of, the study clearly described?

Yes

Are sufficient details of the methods provided to allow replication by others?

Yes

Are the datasets clearly presented in a useable and accessible format?

Not applicable

Reviewer Expertise:

My areas of expertise include: Maternal and Child Nutrition, Food Security and Food systems.

I confirm that I have read this submission and believe that I have an appropriate level of expertise to confirm that it is of an acceptable scientific standard.

# Reviewer report: Reviewer response for version 2

**DOI:** 10.21956/wellcomeopenres.21703.r62959

**Published:** 2023-08-17

**Authors:** Leah Salm

**Affiliations:** 1 Institute of Development Studies, Brighton, England, UK

This is a timely and novel area of research. The mixed-method design appropriately reflects the complex ecological, social, cultural, and health relationships present in this context. This study takes a clear and logical path, and it is underpinned by a trusting and meaningful collaboration with members of the Shawi community which no doubt will ground this research in local understanding, and need which is so vital given the injustices and exclusion indigenous populations have faced.

 I gather this study has already concluded the quantitative component so I will focus my comments on the qual design and analysis. Mostly it is clarifications that I think will help to justify or rationalize the approach.

 1 - The focus is justifiably on Anaemia. I wonder if in the introduction you can also refer to the prevalence of overweight/ obesity or NCDs in this community given that we know low dietary diversity and FBD can also be associated with these burdens of malnutrition. Increasing FBD can be seen as a 'win-win-win' for undernutrition, obesity/ NCD as well as planetary health - so would be interesting to make this link in your context. Related to this it seems vital to understand how connected communities are to food that is not home-grown or wild. Do people stock up on stables from stores? Do people make trips to the city? Are there local vendors for snack foods? Exploration of the food environment literature can help to guide this. This will also be important when thinking through the coping/ adapting/ transforming - it may be people's exchange modality shift e.g. from growing to purchasing, or more swapping, or relying on friends and family for food. Bogard et al categorize these in a useful way. https://www.mdpi.com/2304-8158/10/11/2592

  2 - For the quant study (linked to the above comment) -  Figure 4 - the assumption is that FBD is only affected by flooding. As I see it access socio-economic factors will also determine this - eg a cash aid program may increase HH income and ability to purchase seeds to grow more diverse crops or acquire foods by other means (eg purchase). I wonder if it is possible to separate FBD into that of gathered/ hunted/ grown and sources elsewhere - maybe this is something you already know from previous research?

 3 - Figure 2 - I think there is a typo under study #2 should it be qualitative? Furthermore, the colour coding is not clear, a legend would be helpful especially for the yellow and grey along boxes at the bottom - some seem to be tools, others research questions but these don't appear to be consistent.

 Figure 3 - Consider clarifying - it is not clear what A, B, and C is referring to - what is B.2.1?

 4 - When it comes to food resilience, it is important to acknowledge and explore that coping strategies are not equally distributed - e.g. what are the gendered dimensions? It may be that only women skip meals in the family. May also intersect with other inequitable distribution of outcomes - e.g. those with disabilities, low socio-economic status. If/ where can you capture this?

 We have tried to tease these out in previous research - https://wires.onlinelibrary.wiley.com/doi/10.1002/wcc.696

 5 - It appears there is a comprehensive list for the composition tables, but I wonder are all foods available? What if an item is not, will you look for something similar? We know there can be a lot of variation between varieties even of the same species. Will you be going down to the variety level?

 6 - Figure 5 - Perhaps include more pictorial clues of what less or more resilience looks like - what do the dips signify? Lost crops? Damaged infrastructure? - Would help to bring to life quite abstract terms.

 7 - Figure 6 - What does the wave signify in the high biodiversity category?

 8 - Photovoice (PV) questions - understanding of the words resilient, facilitation, nutrition - are these culturally appropriate and have a shared understanding? I wonder if the interview analysis themes of coping/ adapting/ transforming would be more widely understood as broad prompts? These could be swapped with the more specific questions, with these being added to the interviews instead.

 9 - Will all 28 be in the workshop at the same time?  I believe Wang's rule of thumb in 8-10 for PV workshops. From my experience with this methodology is takes a lot of time  for everyone to speak about their photos and you want everyone to feel heard. Presenting to a smaller group may make people feel more comfortable to open up a little more. Or if time is a limiting factor, there could be two groups working in parallel.  I would also suggest the consideration of dissemination to policy makers to occur after the 2nd workshop when everyone has taken their photos and shared them - rather than during the 1st workshop. The reason is that people will have a better idea of what they are agreeing to, and if decided at the beginning, it might influence what they take pictures of.

 10 - Related to reimbursement to time in taking part. Will participants be given any compensation? Similarly what happens to the cameras, is there an option for them to be kept by community members?

 11 - *"Include a social family and community networks dimension in order to make sense of the long-time relationship among forests, foods and Indigenous people"* Would be good to clarify how this is going to happen. Through the analysis? Through interview questions?

 All the best. I look forward to seeing how these methods come together.

Is the study design appropriate for the research question?

Yes

Is the rationale for, and objectives of, the study clearly described?

Yes

Are sufficient details of the methods provided to allow replication by others?

Partly

Are the datasets clearly presented in a useable and accessible format?

Yes

Reviewer Expertise:

Food systems, food environments, lived experience, food biodiversity

I confirm that I have read this submission and believe that I have an appropriate level of expertise to confirm that it is of an acceptable scientific standard.

## References

[1] : A Typology of Food Environments in the Pacific Region and Their Relationship to Diet Quality in Solomon Islands. *Foods* .2021;10(11) : 10.3390/foods10112592 10.3390/foods10112592 34828873 PMC8620377

[2] : How climate change interacts with inequity to affect nutrition. *WIREs Climate Change* .2021;12(2) : 10.1002/wcc.696 10.1002/wcc.696

# Reviewer report: Reviewer response for version 1

**DOI:** 10.21956/wellcomeopenres.20214.r53491

**Published:** 2023-01-30

**Authors:** Thomas F. Thornton

**Affiliations:** 1 Environmental Change Institute, University of Oxford, Oxford, UK; 2 Alaska Coastal Rainforest Center, University of Alaska Southeast, Juneau, AK, USA

This is an important and worthy topic and the mixed method quantitative/qualitative approach is generally well conceived. There are a lot of assumptions, however, that need further examination, starting with two central concepts: floods and biodiversity.

 It has been established in the ethnographic literature that "flood" and "drought" are relative concepts and that peoples who experience them may hold different cultural models of what they mean and how to respond to them. The same is true of biodiversity which is based on certain categorization on species which may be different from that of Indigenous peoples (the whole subfield of ethnobiology is built around this notion). Despite the promise of 'participatory methods" there is no indication that the study would interrogate its own assumptions or models of floods and biodiversity. Similarly, the proposal lacks a basic description of the subsistence portfolio and seasonal round of the communities, and its not clear how the study would investigate or establish this baseline (which is likely already a flexible one with considerable adaptive capacity, due to environmental variation) from which to evaluate the diversity stress associated with anemia. Some foods also vary in nutrition according to season, maturity, etc. (though perhaps this doesn't affect iron as much). The one month FBD recall period will surely not capture this variability, especially with nearly 150 species at play over an annual cycle (or perhaps multi-year--we don't know from the very limited context provided). 

 Similarly, how has/does the regional economy function in terms of trade for diverse or nutritious items? Modern roads are mentioned as potential access corridors to non-local foods (with cash), but what have been the historical trade relationships? And how have they functioned, and perhaps still function, in times of flood or other environmental stresses? Peruvian communities have long been interconnected in various ways that matter during times of stress. I don't get a sense that community networks and the role they may play in supporting resilience in the wake of temporary local flooding, is going to be investigated. Some innovative work in this areas has been done with Alaska Native communities by S. BurnSilver and colleagues (cf. BurnSilver, S., Coleman, J. and Magdanz, J., 2022. Equality and equity in Arctic communities: how household-level social relations support community-level social resilience. *Ecology and Society*, *27*(3) and references therein. One finding is networks are not uniform among households, but rather can vary widely among households in a community as some have stronger ties to other communities than others, with important implications for local vulnerability and resilience).

 Simple community based participatory methods and photovoice, though laudable, will not necessarily capture the issues above. A more ethnographic approach is warranted that looks at the historical ecological development of the region (c.f. the work of William Balée, e.g., Balée, W., 1998. Historical ecology: premises and postulates. *Advances in historical ecology*, pp.13-29., and Balée, W., 2013. *Cultural forests of the Amazon: a historical ecology of people and their landscapes*. University of Alabama Press.). It is not hard to add this dimension to the study methodology, and much could be accomplished with a strong review of the anthropological literature, and the addition of some thoughtful structured and semi-structured questions around floods, droughts, and intra and inter-community food systems.

 Such an approach will improve both the methodology, data collection, and interpretation of the results in terms of broader health and food system resilience.

Is the study design appropriate for the research question?

Partly

Is the rationale for, and objectives of, the study clearly described?

Yes

Are sufficient details of the methods provided to allow replication by others?

Partly

Are the datasets clearly presented in a useable and accessible format?

Partly

Reviewer Expertise:

Environmental anthropology, environmental change, human adaptation to biodiversity change.

I confirm that I have read this submission and believe that I have an appropriate level of expertise to confirm that it is of an acceptable scientific standard, however I have significant reservations, as outlined above.

## References

[1] : Equality and equity in Arctic communities: how household-level social relations support community-level social resilience. *Ecology and Society* .2022;27(3) : 10.5751/ES-13479-270331 10.5751/ES-13479-270331

[2] : Historical ecology: premises and postulates. *Advances in historical ecology. Columbia University Press, New York* .1998;13-29

[3] : Cultural forests of the Amazon: a historical ecology of people and their landscapes. *University of Alabama Press, Tuscaloosa* .2013;

# Response

**Published:** 2023-06-03

**Authors:** Carol Zavaleta-Cortijo

**Affiliations:** UPCH, Peru

This is an important and worthy topic and the mixed method quantitative/qualitative approach is generally well conceived. There are a lot of assumptions, however, that need further examination, starting with two central concepts: floods and biodiversity.

(*) We are pleased that our reviewer finds our research topic and research approach relevant to the scientific community. We will pass now to address reviewer’s comments and concerns. Please see (*)

 It has been established in the ethnographic literature that "flood" and "drought" are relative concepts and that peoples who experience them may hold different cultural models of what they mean and how to respond to them.

 ( *) We agree that weather characteristics are relative and based on cultural models. We are anticipating navigate this concern by working with Shawi as a collaborators and researchers. Members of our research team are young and adults’ Indigenous people from  Shawi communities: Adult male and female (husband and wife) Shawi community Indigenous leaders (Manuel and Rogelia), one youth male Shawi nursing student (Junior), one youth female Shawi health technician (Nerita), one adult male  Shawi health technician (Guillermo). Neither of them have completed a previous training on environmental or social science. They have provided this study with key information on the definition and Shawi conceptualization of floods and other weather characteristics in their communities. A version in Shawi and Spanish key terminology is attached for your information ( **Extended Materials 2** https://doi.org/10.6084/m9.figshare.23290304.v3 ) . During the fieldwork we plan to validate this concept list with the participants to have a better understanding of how Shawi Indigenous collaborators, understand extreme climatic events.

 The same is true of biodiversity which is based on certain categorization on species which may be different from that of Indigenous peoples (the whole subfield of ethnobiology is built around this notion). Despite the promise of 'participatory methods" there is no indication that the study would interrogate its own assumptions or models of floods and biodiversity.

(*) Definitely to identify and categorize biodiversity is a challenge and depends on the geographical-ecological context. During the cooking workshops, we were able to revise and validate our original list of individual foods and species that families consumed. This list is informing the preparation of our 24-hour recall tool and is helping us to better understand how biodiversity is used by our Shawi collaborators. We have completed four cooking workshops to come out with two comprehensive list of species one for men and one for women. Please see attached lists ( **Extended Materials 3 and 4 https://doi.org/10.6084/m9.figshare.23290562.v2**)

 Similarly, the proposal lacks a basic description of the subsistence portfolio and seasonal round of the communities, and its not clear how the study would investigate or establish this baseline (which is likely already a flexible one with considerable adaptive capacity, due to environmental variation) from which to evaluate the diversity stress associated with anemia. Some foods also vary in nutrition according to season, maturity, etc. (though perhaps this doesn't affect iron as much). The one month FBD recall period will surely not capture this variability, especially with nearly 150 species at play over an annual cycle (or perhaps multi-year--we don't know from the very limited context provided). 

(*) This study was original frame to complete seasonal collection of data, to be able to report on seasonal differences, on anemia prevalence, but also on FBD changes over the year and the qualitative analysis as well. As we have explained in the protocol section of “Adaptations made because of the COVID-19 pandemic”, we were not able to complete seasonal data collection anymore. However, we have completed previous studies with this same population that are helping us to understand seasonal changes that we plan to use to triangulate our results. Also, the **Extended Materials 3 and 4**, have a column with information on how food biodiversity varies over seasons.

 Ref 1: Zavaleta, C., Berrang-Ford, L., Ford, J., Llanos-Cuentas, A., Carcamo, C., Ross, N. A., ... & Indigenous Health and Adaption to Climate Change Research Group. (2018). Multiple non-climatic drivers of food insecurity reinforce climate change maladaptation trajectories among Peruvian Indigenous Shawi in the Amazon. *PLoS One*, *13*(10), e0205714.

 Ref 2: Arotoma-Rojas, I., Berrang-Ford, L., Zavaleta-Cortijo, C., Ford, J. D., & Cooke, P. (2022). Indigenous Peoples’ Percepcions of Their Food System in the Context of Climate Change: A Case Study of Shawi Men in the Peruvian Amazon. *Sustainability*, *14*(24), 16502.

 Ref 3: Valdivia-Gago Andrea, et al. The seasonality of nutrition status in Shawi Indigenous children in the Peruvian Amazon. Currently under revision at PlosOne

 Similarly, how has/does the regional economy function in terms of trade for diverse or nutritious items? Modern roads are mentioned as potential access corridors to non-local foods (with cash), but what have been the historical trade relationships? And how have they functioned, and perhaps still function, in times of flood or other environmental stresses? Peruvian communities have long been interconnected in various ways that matter during times of stress. I don't get a sense that community networks and the role they may play in supporting resilience in the wake of temporary local flooding, is going to be investigated. Some innovative work in this areas has been done with Alaska Native communities by S. BurnSilver and colleagues (cf. BurnSilver, S., Coleman, J. and Magdanz, J., 2022. Equality and equity in Arctic communities: how household-level social relations support community-level social resilience. *Ecology and Society*, *27*(3) and references therein. One finding is networks are not uniform among households, but rather can vary widely among households in a community as some have stronger ties to other communities than others, with important implications for local vulnerability and resilience).

(*) We are grateful to the reviewer to provide these rich literature to inform our research  on trade and other type of networks supporting  food security among Indigenous communities. During the Photovoice, participants will be invited to reflect on how networks within and outside the community have supported their food security in previous extreme floodings. We are going to add this dimension also to the semi-structured interviews as a complementary topic to investigate the contribution of external and within community networks, specially during extreme climatic events. To our knowledge the external aid has been marginal, not really providing the support after an extreme climatic event. These findings are part of our preliminary approach when we contact the leaders of the communities. **(Extended materials 5 for protocol https://doi.org/10.6084/m9.figshare.23290601.v1**)

 Simple community based participatory methods and photovoice, though laudable, will not necessarily capture the issues above. A more ethnographic approach is warranted that looks at the historical ecological development of the region (c.f. the work of William Balée, e.g., Balée, W., 1998. Historical ecology: premises and postulates. *Advances in historical ecology*, pp.13-29., and Balée, W., 2013. *Cultural forests of the Amazon: a historical ecology of people and their landscapes*. University of Alabama Press.). It is not hard to add this dimension to the study methodology, and much could be accomplished with a strong review of the anthropological literature, and the addition of some thoughtful structured and semi-structured questions around floods, droughts, and intra and inter-community food systems.

 Such an approach will improve both the methodology, data collection, and interpretation of the results in terms of broader health and food system resilience.

(*) We agree and humbly accepted the reviewer comments and thoughtful suggestions to improve our work. These new references and also deeply exploration and analysis of the weather and biodiversity understanding; and also, how external or internal networks  support  food security among Indigenous communities, will be added in the qualitative component, since we are right now heading to that step. *We also want to thank to the reviewer for their patience in waiting for our answers, we were hoping to have more comments (other reviewers) before to proceed to answer, however, since we are now finishing the analysis of the  quantitative part, and planning the qualitative component, we consider is the right time to comment to our reviewer that we are taking account their suggestions.* *We hope our comments, and additional Extended materials  (2, 3,  4 and 5) will clarify our assumptions and satisfy the concerns of our reviewer.* *All best* *BioKusharu team*
